# Impact of Varied Factors on Iron, Nickel, Molybdenum and Vanadium Concentrations in the Knee Joint

**DOI:** 10.3390/ijerph17030813

**Published:** 2020-01-28

**Authors:** Karolina Kot, Danuta Kosik-Bogacka, Paweł Ziętek, Maciej Karaczun, Żaneta Ciosek, Natalia Łanocha-Arendarczyk

**Affiliations:** 1 Department of Biology and Medical Parasitology, Pomeranian Medical University in Szczecin, Powstancow Wielkopolskich 72, 70-111 Szczecin, Poland; kotkarolina17@gmail.com (K.K.); nlanocha@pum.edu.pl (N.Ł.-A.); 2 Independent of Pharmaceutical Botany, Department of Biology and Medical Parasitology, Pomeranian Medical University in Szczecin, Powstancow Wielkopolskich 72, 70-111 Szczecin, Poland; 3 Chair and Clinic of Orthopaedics, Traumatology and Oncology, Pomeranian Medical University in Szczecin, Unii Lubelskiej 1, 71-252 Szczecin, Poland; paulz@wp.pl (P.Z.); dooogie@tlen.pl (M.K.); 4 Laboratory of Medical Rehabilitation, Pomeranian Medical University in Szczecin, Zolnierska 54, 71-210 Szczecin, Poland; ciosekzaneta@gmail.com

**Keywords:** trace elements, spongy bone, cartilage, meniscus, anterior cruciate ligament, infrapatellar fat pad

## Abstract

The aim of this study was to determine the concentrations of iron, nickel, molybdenum, and vanadium in the knee joint. We also examined the relationships between the concentrations of these metals in the knee joint and the influence of varied factors on the concentration of Fe, Ni, Mo, and V. The study of these trace elements is important, because these elements are used alone and in combination in diet supplements, and they are components of biomaterials implanted in medicine. The study materials, consisting of the spongy bone, cartilage, meniscus, anterior cruciate ligament (ACL), and infrapatellar fat pad, were obtained from 34 women and 12 men from northwestern Poland. The concentrations of Ni, Fe, Mo, and V were determined using spectrophotometric atomic absorption in inductively coupled argon plasma (ICP-AES). We found significantly higher Mo concentrations in the ACL of women than men. There was a significant difference in the Mo concentration in the spongy bone between patients from cities with fewer than 100,000 inhabitants and patients from cities with more than 100,000 residents. Iron concentrations in the spongy bone were higher in non-smoking patients and those who did not consume alcohol. Vanadium concentrations were higher in the infrapatellar fat pads in abstainers. In patients who had not undergone arthroscopy surgery, V concentration was lower in cartilage. The concentrations of V in the cartilage and infrapatellar fat pad were higher in osteoporotic patients than in non-osteoporotic patients. There were significant differences in Fe concentrations in the meniscus, with the lowest in osteoporotic patients. We noted lower Mo concentrations in the spongy bone of patients with rheumatoid arthritis. Furthermore, we noted some new interactions among metals in the studied structures of the knee joint. The results reported in this study show the influence of gender, place of residence, smoking, consumption of alcohol, arthroscopy surgery, osteoporosis, and rheumatoid arthritis on the Fe, Ni, Mo, and V concentrations in the studied structures of the knee joint.

## 1. Introduction

The skeleton provides the body with its structure and also functions in hematopoietic processes, mineral composition, and a variety of other physiological processes [1]. Bone tissue is constantly undergoing the process of bone turnover in which osteoclasts resorb bone and osteoblasts fill the resorptive pits with new bone. Bone tissue has a long remodeling time, so it can reflect long-term exposure to toxic metals. It can also be used as a biomarker for indirect assessment of metal accumulation from the environment [2]. Many elements have a significant effect on bone health and metabolism [3]. It has been shown that a change in the composition of elements in the bone and cartilage tissue can cause degenerative changes in joints [4]. 

Iron and nickel are essential elements for many animal species, microorganisms, plants, and in humans. Iron is involved in collagen synthesis and the conversion of 25-hydroxy vitamin D to its active form [5,6,7]. Available studies have presented that Fe deficiency can disturb bone homeostasis. Both bone formation and resorption are affected, causing decreased bone mineral density and mass, altered microarchitecture, and reduced strength [8,9,10]. High Fe concentration is associated with disturbed metabolism in both osteoblasts and osteoclasts. In osteoblasts, Fe overload exerts antagonistic actions on pre-osteoblast cells, disrupting cell differentiation [11,12,13]. Moreover, Fe overload may be involved in different types of osteoporosis, incoming bone resorption and oxidative stress, decreasing bone biomechanical properties, cartilage degeneration, and increasing the risk of fracture [12,14,15,16,17]. The role of Ni in the physiology of the human body is not well understood [18]. Nickel is mainly found in soft tissues; however, its presence in the osseous tissue has also been confirmed. This element influences the skeleton metabolism [18,19,20]. *In vitro* studies have shown that excess Ni concentration inhibits the activity of alkaline phosphatase and, consequently, leads to bone mineralization. It has been shown that Ni has a cytotoxic effect on osteocytes in culture inducing cell apoptosis [21,22]. No reports on the effects of Ni poisoning on human bone are presented in the literature [23]. Molybdenum is a cofactor for several redox enzymes. In the human body, Mo accumulates mostly in the liver, kidneys, and bones [24]. Molybdenum deficiency and overload are uncommon in human populations. Experiments conducted on animals have shown that Mo deficiency can inhibit growth, especially in early stages of development [25]. High Mo concentration is toxic and may cause bone deformities similar to the changes occurring in the rheumatoid arthritis, tooth decay, and lipid and protein disorders [19]. It is suggested that other direct actions of Mo exert on bone metabolism, but the mechanisms are still unknown and need to be explored [26]. Vanadium is necessary for metabolic processes including transformations of lipids, phospholipids, and cholesterol [27,28,29,30]. Vanadate, a known phosphotyrosyl protein phosphatase inhibitor, stimulates bone formation by increasing the number of mature osteoblasts [31]. Supplementation with V stimulates osteogenic cell proliferation and collagen production as well as increases bone mineral density, mineralization, and formation [32,33]. On the other hand, excess V may cause biochemical and hematological changes resulting in neurological damage and function impairment of the bones, kidneys, liver, and spleen [34]. In humans, approximately 50% of V accumulates in the bones, with the remaining part in the kidneys, spleen, liver, blood, adipose tissue, and brain [35]. 

In our previous study, we observed the influence of some parameters (e.g., age, gender, BMI, and hypertension) on element concentrations in the knee joint [36,37]. Therefore, the aim of this study was to determine Fe, Mo, Ni, and V concentrations in the spongy bone, cartilage, meniscus, anterior cruciate ligament (ACL), and infrapatellar fat pad. Another objective was to examine the relationships between the concentrations of these metals in the studied parts of the knee joint and the influence of gender, age, place of residence, smoking, alcohol consumption, having an endoprosthesis, undergoing arthroscopy, and coexisting diseases (i.e., hypertension, osteoporosis, rheumatoid arthritis, and diabetes) on the concentrations of the studied elements in parts of the knee joint. The study of these trace elements are important, because these elements are used alone and in combination in diet supplements, and they are components of biomaterials implanted in medicine.

## 2. Material and Methods

The study was approved by the Bioethics Committee of the Pomeranian Medical University in Szczecin (KB-0012/56/14). This study conformed to the principles outlined in The Declaration of Helsinki as revised in 2008.

The materials were obtained from 46 patients including 34 women aged 56–87 (73.1 ± 8.2 years) and 12 men aged 59–85 (73.5 ± 8.3 years) from northwestern Poland. The samples were collected in patients from the Chair and Clinic of Orthopedics and Traumatology at the Pomeranian Medical University in Szczecin. 

We examined 44 samples of spongy bone and 46 samples of the cartilage, meniscus, ACL as well as infrapatellar fat pads removed from 46 patients following knee arthroplasty. The patients were interviewed concerning demographics and health status. The patients participating in the study were informed about the course of the research project and provided written consent prior to participation.

The samples were dried to 105 °C and wet digested in a mixture of concentrated 65% nitric acid (Suprapur Merck^®^, Germany) and 30% hydrogen peroxide (Baker Analyzed, Phillipsburg, NJ, USA). The concentrations of Ni, Fe, Mo, and V were determined using spectrophotometric atomic absorption in inductively coupled argon plasma (ICP-AES), on a Perkin–Elmer Optima 2000 DV (Norwalk, CT, USA). More analytical procedures are presented by Ciosek et al. [38]. The concentrations of elements were expressed as mg/kg dry weight (dw). 

The procedures were validated with certified reference material—NIST 8414 Bovine Muscle (National Institute of Standards and Technology). The concentration values of the reference materials given by the manufacturers and our determinations are shown in Table 1.

Statistical analysis was performed using Statistica PL software 10.0 and Microsoft Excel 2016. The distribution normality was examined using a Shapiro–Wilk test. The arithmetic mean (AM), standard deviation (SD), median (Med) as well as minimum and maximum ranges were calculated. Intergroup comparisons were performed using the Mann–Whitney U test and Kruskal–Wallis test. The correlations among the studied metals in the parts of the knee joint were analyzed on the basis of Spearman’s correlation factor (*r*_s_). The significance level was *p* < 0.05. 

## 3. Results

Concentrations of Fe, Ni, Mo, and V in the spongy bone, cartilage, meniscus, anterior cruciate ligament, and infrapatellar fat pad are presented in Table 2. Iron had the highest concentration, while V the lowest concentration in almost all of the analyzed structures of the knee joint. We observed differences in the concentration of the studied elements in the parts of the knee joint. With respect to gender, Mo concentration in the ACL was statistically significantly different, approximately 30% lower in men than in women. Taking into consideration the age of the participants, we did not find any statistical differences between patients below and above 70 years of age. 

There were significant differences in the Mo concentration in the spongy bone between patients from cities with fewer than 100,000 inhabitants and patients from cities with more than 100,000 inhabitants (U = 45, *p* = 0.02), at 0.800 and 0.980 mg/kg dw, respectively. 

There were statistically significant differences in Fe concentrations in spongy bone between non-smoking (NS) and smoking (S) patients. We found above >1.7 times higher Fe concentration in the spongy bone obtained from NS than S patients (60.130 and 34.370 mg/kg dw, respectively). However, for S patients, there were only 7 participants which could have strongly affected the outcome of the comparisons. Iron concentrations in the cartilage, ACL, and infrapatellar fat pads of the NS patients were higher than in the S patients at 76.851 versus 48.146, 92.494 versus 74.603, and 54.128 versus 28.027 mg/kg dw, respectively. The maximum Ni concentration was recorded in the meniscus of S patient (71.378 mg/kg dw). In S patients, the meniscus Mo concentration was approximately two times higher than in NS patients: 89.891 and 44.471 mg/kg dw, respectively. A comparison of the mean levels of elements showed statistically significant differences in the Fe and V concentrations between the patients who consumed alcohol (A; *n* = 7) and those who did not (NA; *n* = 39). We observed higher Fe concentrations in the spongy bones of NA than A patients at 61.293 and 28.224 mg/kg dw, respectively (U = 39, *p* < 0.01). The concentrations of V in the infrapatellar fat pads were statistically significantly different between NA and A patients (U = 69.5, *p* < 0.05) at 0.081 and 0.054 mg/kg dw, respectively. 

Vanadium concentration was significantly higher in patients after arthroscopy surgery (AS; *n* = 4). In the group of patients who had not undergone arthroscopy surgery (NAS; *n* = 42) V concentration was lower in the cartilage than in AS patients, 0.025 and 0.034 mg/kg dw (U = 26, *p* = 0.02). There were differences in Mo concentrations in the meniscus and infrapatellar fat pads between AS and NAS patients, but the differences were not statistically significant. We observed higher Mo concentration in the meniscus in AS (114.733 mg/kg dw) than in the NAS patients (45.349 mg/kg dw), while in the infrapatellar fat pads, Mo concentrations were higher in the NAS group (16.424 mg/kg dw) than in the AS group (2.175 mg/kg dw). 

Taking into account bone diseases, there were significant differences in the V concentration in the cartilage, meniscus, and infrapatellar fat pads between patients with osteoporosis (O; *n* = 3) and without osteoporosis (NO; *n* = 43) (U = 10, *p* < 0.01; U = 15, *p* < 0.05 and U = 14.5, *p* = 0.02, respectively). The concentrations of V in the cartilage and infrapatellar fat pads in the O group were higher than in the NO group at 0.038 versus 0.024 and 0.090 versus 0.076 mg/kg dw. However, V concentrations in the meniscus were higher in NO (0.103 mg/kg dw) compared to O patients (0.063 mg/kg dw). There was a significant difference in the Fe concentration in the meniscus with the lowest in the O group (14.836 mg/kg dw) and the highest in NO group (39.790 mg/kg dw). We noted lower Mo concentrations in the spongy bone of patients with rheumatoid arthritis (RA, *n* = 4) compared to patients without rheumatoid arthritis (NRA, *n* = 42) at 0.403 and 0.998 mg/kg dw, respectively (U = 29, *p* = 0.03). 

A comparison of the mean levels of chemical elements showed no statistically significant differences among patients with hypertension or diabetes compared to healthy participants. Additionally, we did not observe any differences in metal levels in the structures forming knee joints between patients with and without endoprosthesis. 

Pearson’s correlation coefficients between Fe, Ni, Mo, and V in the analyzed parts of the knee joint are presented in Table 3.

## 4. Discussion

Changed deposition of trace elements in tissues or deposition of metals in the form of compounds with low activity may be involved in biochemical processes leading to some diseases. Osteoarthritis (OA), which is a degenerative joint disease, is accompanied by imbalances in trace elements concentration, not only in the bones and cartilage of the joint but also in the ligaments and meniscus [39,40]. Therefore, the metal content of the meniscus and ligaments is important for maintaining strength and elasticity and hence proper functioning of the joint. 

Structures in the knee joint differ in the mineral composition. Most of the Ni, Mo, and V concentrations were in the meniscus and anterior cruciate ligament. This means that most of these elements accumulated in the connective tissues rather than in the bone or cartilage tissue. Roczniak et al. [4] also noted that there is ~50% more Ni concentration in the meniscus compared to the tibia and femur. It has been shown that Mo and Ni may contribute to lipid peroxidation and oxidative stress [41,42] which, according to recent studies, is significantly related to OA progression [43]. Nickel is probably absorbed through transport with interaction of Fe found in the hemoglobin and participates in oxygen transport [44,45]. In our study, statistically significant Fe–Ni correlations were observed in the spongy bone, cartilage, and infrapatellar fat pad. Dąbrowski et al. [45] also noted significantly positive Fe/Ni interaction in the bone. 

In the present study, diverse values of Fe in the analyzed samples of the joint were noted which is likely to have resulted from ongoing degenerative changes in the knee joint. Zaichick and Zaichick [46] noted that the mean physiological Fe concentration in the bones of individuals from Central Europe was 55.5 mg/kg dw. In the spongy bone of the patients from northwestern Poland, the Fe concentration was 56.032 mg/kg dw. Łanocha et al. [47] and Budis et al. [48] found lower Fe level in patients from the same area (43.9 and 43 mg/kg dw, respectively). The difference may result from the different study materials. The spongy bone in our study were obtained from the knee joint, and in the studies by Łanocha et al. [47] and Budis et al. [48] they were taken from the hip joint. In respect to the gender of the participants, we did not observe statistically significant differences in Fe concentration in studies of the knee joint. Budis et al. [48] found similar Fe concentrations in the cartilage and spongy bone in men and women. Brodziak-Dopierała et al. [49] also noted no statistically significant differences in Fe concentration in the meniscus between men and women. Some researchers have observed differences in Fe concentration between older and younger patients; however, those differences are usually slight [46,50,51]. In our study, as in the study by Budis et al. [48], no significant differences in Fe concentration were found between the two age groups. 

Nickel concentration in bones varied even in the same geographical location. Brodziak-Dopierała et al. [20] observed higher Ni concentration in the spongy bone (8.39 mg/kg dw) than in our study (4.75 mg/kg dw). However, in other studies by the same authors, the Ni concentration in the cancellous bone was 4.56 mg/kg dw [52]. Roczniak et al. [4], investigating the residence of the same area as Brodziak-Dopierała et al. [20,51], observed much lower Ni concentrations in the tibia, femur, and meniscus at 0.52, 0.60, and 0.85 mg/kg dw, respectively. Zioła-Frankowska et al. [53], in patents from Wielkopolska (Poland), noted that Ni concentrations were 0.6 mg/kg dw in the femoral head and 0.79 mg/kg dw in the femoral neck. Łanocha-Arendarczyk et al. [54] found even lower Ni concentrations in the cartilage and spongy bone of the hip joint at 0.206 and 0.379 mg/kg dw, respectively. It is important to mark that Łanocha-Arendarczyk et al. [54] examined patients from the West Pomeranian district where the patients from our study live. Kubaszewki et al. [55] found that Ni concentrations in the femoral bone were found to be related with male sex, where osteoporotic changes are observed later than in females. In our study, similar to Łanocha-Arendarczyk et al. [54] and Brodziak-Dopierała et al. [52], no significant differences in Ni concentration were found between men and women and among age groups.

The average Mo concentration in the skeleton of the reference man is <0.48 mg/kg [56]. Kubaszewki et al. [55] found a higher Mo concentration in the bone, ranging from 0.36 to 1.9 mg/kg dw. Zioła-Frankowska et al. [53] observed that the median Mo concentration in the femoral head and neck was 0.18 mg/kg dw. In our study, we noted higher Mo concentrations in the spongy bone of the knee joint, ranging 0.021 to 2.377 mg/kg dw, with a mean value of 0.935 mg/kg dw. Taking into account the gender of the patients, we observed higher Mo concentrations in the anterior cruciate ligament in women than in men. It has been shown that, in women, Mo is released from the bones along with the increasing demineralization of bones with age. A potential Mo mechanism of action is the effect on the hormonal system by disruption of sex hormones and, indirectly, on bone health [57].

Little is known about V concentration in the structures of the knee joint. Łanocha-Arendarczyk et al. [54] found higher V levels in the cartilage and spongy bone of the hip joint (at 0.42 and 0.50 mg/kg dw) compared to presented studies (0.025 and 0.022 mg/kg dw, respectively). The authors did not find statistically significant gender-related differences in V concentrations in the spongy bone and cartilage. In our study, we also did not notice differences in V concentrations in the studied part of the knee joint between female and male patients. 

Smoking affects the distribution of elements in different parts of the knee joint and adversely affects bone density [58]. Tobacco components reduce bone density by inhibiting the formation of osteoblasts as well as apoptosis in other cell types [59]. According to a Surgeon General’s report [60], tobacco smoking is linked with several skeletal system disorders such as fractures and rheumatoid arthritis. Smoking can have a significant impact on the amount of Ni absorbed by the body; therefore, smokers are more vulnerable to its adverse effects than non-smokers. Even passive smoking increases Ni accumulation in the tissues [61]. Brodziak-Dopierała et al. [20] also confirmed that the habit of tobacco smoking might be the causative agent of elevated content of Ni in the osseous tissue. The authors showed that the Ni concentration in the femoral head of smokers was higher than in non-smokers at 6.09 and 4.52 mg/kg dw, respectively. Łanocha-Arendarczyk et al. [54] also observed higher Ni concentrations in the articular cartilage and cancellous bone in smokers (0.22 versus 0.41 mg/kg dw) compared to non-smoking patients (0.19 versus 0.32 mg/kg dw). However, the significant differences in Ni concentrations were demonstrated only for cartilage. Different results are presented by Kwapulinski et al. [61] and Roczniak et al. [4], who showed that Ni concentration was higher in non-smokers than smokers. In our study, we also observed higher Ni concentrations in the spongy bone, cartilage, ACL, and infrapatellar fat pad of non-smoking than smoking patients; however, no differences in Ni content were statistically significant. In the present study, there was a statistically significant difference in Fe concentration in the spongy bone. We noted higher Fe levels in the non-smoking group of patients (60.130 mg/kg dw) than in the smoking group (34.375 mg/kg dw). Brodziak-Dopierała et al. [52] also observed higher Fe contents in the knee joints of non-smokers (39.11 mg/kg dw) compared to smokers (25.47 mg/kg dw). 

It has been observed that ethanol may decrease bone formation and increase its resorption, resulting in bone demineralization. Moreover, ethanol can have a modulating effect through mineral regulating hormones such as vitamin D metabolites, parathyroid hormone, and calcitonin [62,63]. Zioła-Frankowska et al. [53] found statistically significant differences between Mo and Ni concentrations in the cancellous bone of the hip joint according to alcohol consumption. They observed higher Ni and lower Mo concentrations in the spongy bone of patients who consumed alcohol compared to abstainers. In our study, we observed statistically lower Fe concentration in the spongy bone of patients who consumed alcohol compared to patients who did not at 28.224 and 61.293 mg/kg dw, respectively. Zioła-Frankowska et al. [53] also found lower Fe level in the cancellous bone of patients consuming alcohol; however the difference was not statistically significant. 

Trace element concentration in the structures building the knee joint may be influences by endoprostheses. Research on metal release from implants showed the presence of chromium and nickel in tissues surrounding the implant. Łanocha-Arendarczyk et al. [54] found higher Ni concentration in the articular cartilage and cancellous bone in patients who had implants (0.34 and 0.42 mg/kg dw) compared to patients without implants (0.18 and 0.26 mg/kg dw), yet statistically significant differences in concentrations were recorded only for the cancellous bone. In the present study, we found higher Ni level in the structures of the knee joint of patients with implants (ranging 0.02–98.19 mg/kg dw) compared to patients without implants (ranging 0.02–43.89 mg/kg dw); however, no statistically significant differences were observed. We noted, as Brodziak-Dopierała et al. [51], lower Fe contents in patients who had never undergone endoprosthesoplasty than in patients who had already had that type of surgery. However, the differences were not statistically significant. Additionally, we also observed statistically significant higher V concentrations in the cartilage of patients who underwent arthroscopy surgery. It is possible that higher V values resulted from using stainless steel during surgery. 

Bone diseases, including osteoarthritis, osteoporosis, and rheumatoid arthritis, may be accompanied by normal, elevated, or reduced trace element concentration [39]. Noor et al. [64] observed higher V concentrations in OA that without OA patients. In our study, we observed statistically significant higher V concentrations in the cartilage and infrapatellar fat pad of patients with osteoporosis. In a previous study, we observed a negative correlation between Ca and V in the structures of the joint [65]. This relationship may be significant in the development of osteoporosis, because V can substitute for Ca in hydroxyapatite crystals [33]. We also noted statistically significant higher Fe concentrations in the meniscus of non-osteoporotic patients. Budis et al. [48], in osteoporotic patients from northwestern Poland, found higher Fe concentrations in the bone of hip joints, but the difference was not statistically significant. It is important to mark that we cannot compare these results on account of different studied materials. We observed higher Mo concentrations in the spongy bone of patients without rheumatoid arthritis (0.988 mg/kg dw) than in patients suffering from rheumatoid arthritis (0.403 mg/kg dw) (*p* < 0.05). 

Imbalances in trace element metabolism may lead to metal interactions with potential pathophysiological significance. Knowledge of these relationships is important for the understanding of kinetic interactions of elements in the body [66]. Zioła-Frankowska et al. [53] did not observed a correlation between Ni–Mo, Ni–Fe, and Fe–Mo in the femoral neck and head, whereas Dąbrowski et al. [45] noted significantly positive Fe/Ni interaction in the bone. This research is the first to find a correlation among elements in the structures of the knee joint: Fe_SB_–Fe_ACL_, Fe_SB_–Ni_SB_, Fe_C_–Fe_M_, Fe_C_–Ni_C_, Fe_M_–Fe_ACL_, Fe_M_–Fe_IFP_, Fe_M_–Ni_C_, Fe_IFP_–Ni_IFP_, Fe_IFP_–Mo_ACL_, Fe_IFP_–Mo_IFP_, Fe_IFP_–V_IFP_, Ni_SB_–Ni_IFP_, Ni_SB_–Mo_SB_, Ni_C_–Ni_IFP_, Ni_C_–Mo_SB_, Ni_C_–Mo_C_, Ni_M_–Mo_M_, Ni_M_–Mo_ACL_, Ni_M_–V_M_, Ni_ACL_–Mo_ACL_, Ni_ACL_–V_ACL_, Ni_IFP_–Mo_SB_, Ni_IFP_–Mo_C_, Ni_IFP_–Mo_IFP_, Ni_IFP_–V_IFP_, Mo_C_–V_C_, Mo_M_–Mo_ACL_, Mo_M_–V_M_, Mo_ACL_–V_M_, Mo_ACL_–V_ACL_.

## 5. Conclusions

The result reported here may provide a basis for establishing reference values for the spongy bone, cartilage, meniscus, anterior cruciate ligament, and infrapatellar fat pad of patients with osteoarthritis. Analysis of the impact of varied factors on Fe, Ni, Mo, and V concentrations in the knee joint showed the influence of gender, place of residence, smoking, drinking alcohol, arthroscopy surgery, osteoporosis, and rheumatoid arthritis. Moreover, new types of interactions were noted. 

## Figures and Tables

**Table 1:** Analysis of NIST (National Institute of Standards and Technology) 8414 Bovine Muscle.

Chemical Elements	NIST 8414 (Bovine Muscle)		
	Certified	Measured (*n* = 9)	Recovery (%)
Fe	71.20 ± 9.20	64.27 ± 2.45	90%
Ni	0.05 ± 0.04	0.04 ± 0.01	80%
Mo	0.08 ± 0.06	0.10 ± 0.02	125%
V	0.005	0.0045 ± 0.005	90%

**Table 2:** The concentrations of iron, nickel, molybdenum, and vanadium in the spongy bone, cartilage, meniscus, anterior cruciate ligament, and infrapatellar fat pad in women and men (*n*, number of samples; AM, arithmetic mean; SD, standard deviation; Med, median; K–W test, Kruskal–Wallis test; U, Mann–Whitney U test; *p*, level of significance; NS, non-significant difference; concentrations expressed in mg/kg dry weight).

	Concentration of Elements Expressed as mg/kg dw			
	Fe	Ni	Mo	V
Total (*n* = 46)				
*Spongy bone (n = 44)*				
AM ± SD	56.032 ± 37.011	4.752 ± 14.600	0.935 ± 0.505	0.022 ± 0.001
Med	44.885	1.920	0.928	0.022
Range	14.191–169.957	0.018–98.180	0.021–2.377	0.020–0.028
*Cartilage (n = 46)*				
AM ± SD	72.483 ± 63.779	3.909 ± 3.895	1.918 ± 5.541	0.025 ± 0.009
Med	55.086	2.405	1.014	0.022
Range	10.444–393.624	0.020–19.402	0.020–38.419	0.020–0.067
*Meniscus (n = 46)*				
AM ± SD	38.163 ± 28.686	18.708 ± 14.291	51.383 ± 46.872	0.101 ± 0.059
Med	29.592	14.970	41.182	0.082
Range	7.792–166.180	0.080–71.738	0.063–274.449	0.052–0.338
*Anterior cruciate ligament (n = 46)*				
AM ± SD	89.771 ± 57.648	15.833 ± 8.998	42.410 ± 26.214	0.086 ± 0.030
Med	72.919	14.984	43.393	0.086
Range	23.598–243.982	0.075–43.887	0.280–104.450	0.045–0.163
*Infrapatellar fat pad (n = 46)*				
AM ± SD	50.156 ± 59.632	8.310 ± 13.796	7.498 ± 14.621	0.077 ± 0.067
Med	30.612	2.245	2.166	0.065
Range	12.530–367.259	0.034–82.599	0.051–58.665	0.034–0.505
K–W test				
H	45	81	118	164
*p*	<0.01	<0.01	<0.01	<0.01
Female (*n* = 34)				
*Spongy bone (n = 32)*				
AM ± SD	55.004 ± 32.727	2.338 ± 2.338	0.871 ± 0.497	0.022 ± 0.001
Med	51.708	1.492	0.822	0.021
Range	14.191–157.291	0.018–7.360	0.021–2.377	0.020–0.024
*Cartilage (n = 34)*				
AM ± SD	72.217 ± 70.332	3.559 ± 4.089	2.104 ± 6.349	0.025 ± 0.009
Med	51.848	1.897	0.991	0.022
Range	10.444–393.624	0.020–19.402	0.021–38.419	0.020–0.067
*Meniscus (n = 34)*				
AM ± SD	37.374 ± 31.420	18.707 ± 15.326	53.566 ± 49.187	0.100 ± 0.063
Med	26.117	14.185	43.244	0.080
Range	7.792–166.180	0.088–71.738	0.063–274.449	0.052–0.338
*Anterior cruciate ligament (n = 34)*				
AM ± SD	88.186 ± 58.304	16.436 ± 9.407	46.214 ± 27.829	0.089 ± 0.032
Med	66.169	14.832	47.489	0.086
Range	23.598–230.160	0.087–43.887	0.280–104.450	0.045–0.163
*Infrapatellar fat pad (n = 34)*				
AM ± SD	43.875 ± 37.787	5.794 ± 7.545	7.372 ± 15.292	0.070 ± 0.017
Med	30.612	1.689	2.224	0.070
Range	12.593–197.711	0.034–27.823	0.051–58.665	0.034–0.097
Male (*n* = 12)				
*Spongy bone (n = 12)*				
AM ± SD	58.774 ± 45.189	11.187 ± 26.336	1.105 ± 0.466	0.023 ± 0.002
Med	38.345	3.688	1.262	0.022
Range	18.805–169.957	0.021–98.180	0.480–1.928	0.020–0.028
*Cartilage (n = 12)*				
AM ± SD	73.236 ± 35.188	4.903 ± 2.864	1.391 ± 0.746	0.023 ± 0.005
Med	68.112	6.496	1.739	0.021
Range	26.987–146.683	0.081–8.353	0.020–2.168	0.020–0.039
*Meniscus (n = 12)*				
AM ± SD	40.398 ± 16.790	18.713 ± 10.021	45.198 ± 36.501	0.102 ± 0.041
Med	35.061	20.759	41.182	0.096
Range	16.844–70.850	0.080–38.693	0.414–105.537	0.065–0.222
*Anterior cruciate ligament (n = 12)*				
AM ± SD	94.263 ± 52.949	14.126 ± 6.993	31.633 ± 15.014	0.078 ± 0.017
Med	52.949	15.264	38.677	0.081
Range	45.687–243.982	0.075–23.409	0.479–49.297	0.053–0.102
*Infrapatellar fat pad (n = 12)*				
AM ± SD	67.952 ± 94.132	15.439 ± 21.993	7.888 ± 12.306	0.098 ± 0.124
Med	33.608	11.044	2.166	0.057
Range	12.529–367.259	0.036–82.599	0.474–39.848	0.036–0.505
Female vs. Male				
*Spongy bone*				
U	NS	NS	NS	NS
*p*				
*Cartilage*				
U	NS	NS	NS	NS
*p*				
*Meniscus*				
U	NS	NS	NS	NS
*p*				
*Anterior cruciate ligament*				
U	NS	NS	119	NS
*p*			0.03	
*Infrapatellar fat pad*				
U	NS	NS	NS	NS

**Table 3:** Pearson’s correlation between iron, nickel, molybdenum, and vanadium in the analyzed samples (SB, spongy bone; C, cartilage; M, meniscus; ACL, anterior cruciate ligament; IFP, infrapatellar fat pad; NS, non-significant).

		SB				C				ACL				M				IFP			
		Fe	Ni	Mo	V	Fe	Ni	Mo	V	Fe	Ni	Mo	V	Fe	Ni	Mo	V	Fe	Ni	Mo	V
SB	Fe	-																			
	Ni	0.37	-																		
	Mo	NS	0.37	-																	
	V	NS	NS	NS	-																
C	Fe	NS	NS	NS	NS	-															
	Ni	NS	NS	0.37	NS	0.74	-														
	Mo	NS	NS	NS	NS	NS	0.31	-													
	V	NS	NS	NS	NS	NS	NS	0.53	-												
ACL	Fe	NS	NS	NS	NS	0.66	0.40	NS	NS	-											
	Ni	NS	NS	NS	NS	NS	NS	NS	NS	NS	-										
	Mo	NS	NS	NS	NS	NS	NS	NS	NS	NS	0.90	-									
	V	NS	NS	NS	NS	NS	NS	NS	NS	NS	0.91	0.85	-								
M	Fe	0.37	NS	NS	NS	NS	NS	NS	NS	0.37	NS	NS	NS	-							
	Ni	NS	NS	NS	NS	NS	NS	NS	NS	NS	NS	NS	NS	NS	-						
	Mo	NS	NS	NS	NS	NS	NS	NS	NS	NS	0.41	0.35	0.35	NS	0.73	-					
	V	NS	NS	NS	NS	NS	NS	NS	NS	NS	NS	NS	NS	NS	0.63	0.50	-				
IFP	Fe	NS	NS	NS	NS	NS	NS	NS	NS	0.38	NS	NS	NS	NS	NS	0.31	NS	-			
	Ni	NS	0.32	0.45	NS	NS	0.46	0.38	NS	NS	NS	NS	NS	NS	NS	NS	NS	0.36	-		
	Mo	NS	NS	NS	NS	NS	NS	NS	NS	NS	NS	NS	NS	NS	NS	NS	NS	0.45	0.42	-	
	V	NS	NS	NS	NS	NS	NS	NS	NS	NS	NS	NS	NS	NS	NS	NS	NS	0.34	0.36	NS	-
